# *N*-*tert*-Butyl-2-(2,6-di­chloro­phen­yl)imidazo[1,2-a]pyrazin-3-amine

**DOI:** 10.1107/S1600536813013640

**Published:** 2013-05-25

**Authors:** Zeenat Fatima, Thothadri Srinivasan, Suman Koorathota, Sathiah Thennarasu, Devadasan Velmurugan

**Affiliations:** a Centre of Advanced Study in Crystallography and Biophysics, University of Madras, Guindy Campus, Chennai 600 025, India; b Organic Chemistry Division, Central Leather Research Institute, Adyar, Chennai 600 020, India

## Abstract

In the title compound, C_16_H_16_Cl_2_N_4_, the imidazole ring mean plane makes a dihedral angle of 70.01 (1)° with the phenyl ring. The Cl atoms deviate by −0.0472 (6) and 0.0245 (8) Å from the plane of their attached benzene ring. In the crystal, mol­ecules are linked *via* pairs of C—H⋯N hydrogen bonds, forming inversion dimers.

## Related literature

For applications of the pyrazine ring system in drug development, see: Du *et al.* (2009 ▶); Dubinina *et al.* (2006 ▶); Ellsworth *et al.* (2007 ▶); Mukaiyama *et al.* (2007 ▶). For background to the fluorescence properties of related compounds, see: Kawai *et al.* (2001 ▶); Abdullah (2005 ▶). For general background to the use of imidazole derivatives as drugs, see: Dooley *et al.* (1992 ▶); Jackson *et al.* (2000 ▶); Banfi *et al.* (2006 ▶). For related structures, see: Ouzidan *et al.* (2011 ▶); Nasir *et al.* (2010 ▶).
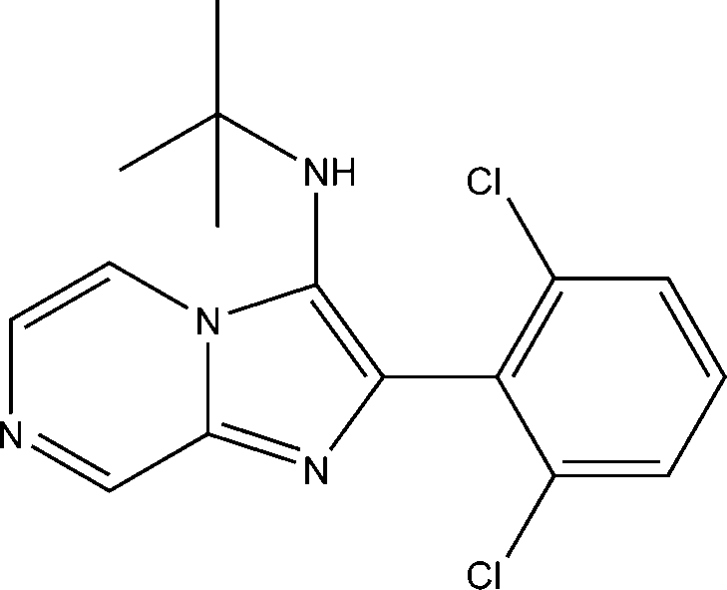


## Experimental

### 

#### Crystal data

C_16_H_16_Cl_2_N_4_
*M* *_r_* = 335.23
Triclinic, 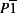

*a* = 8.1482 (4) Å
*b* = 9.8553 (5) Å
*c* = 11.5265 (6) Å
α = 93.218 (2)°
β = 99.320 (3)°
γ = 113.026 (2)°
*V* = 833.31 (7) Å^3^
*Z* = 2
Mo *K*α radiation
μ = 0.39 mm^−1^
*T* = 293 K
0.30 × 0.25 × 0.20 mm

#### Data collection

Bruker SMART APEXII area-detector diffractometer
Absorption correction: multi-scan (*SADABS*; Bruker, 2008 ▶) *T*_min_ = 0.892, *T*_max_ = 0.926
12583 measured reflections
3438 independent reflections
2941 reflections with *I* > 2σ(*I*)
*R*_int_ = 0.026

#### Refinement

*R*[*F* ^2^ > 2σ(*F* ^2^)] = 0.040
*wR*(*F* ^2^) = 0.120
*S* = 1.05
3438 reflections
203 parameters
H-atom parameters constrained
Δρ_max_ = 0.34 e Å^−3^
Δρ_min_ = −0.40 e Å^−3^

### 

Data collection: *APEX2* (Bruker, 2008 ▶); cell refinement: *SAINT* (Bruker, 2008 ▶); data reduction: *SAINT*; program(s) used to solve structure: *SHELXS97* (Sheldrick, 2008 ▶); program(s) used to refine structure: *SHELXL97* (Sheldrick, 2008 ▶); molecular graphics: *ORTEP-3 for Windows* (Farrugia, 2012 ▶); software used to prepare material for publication: *SHELXL97* and *PLATON* (Spek, 2009 ▶).

## Supplementary Material

Crystal structure: contains datablock(s) global, I. DOI: 10.1107/S1600536813013640/su2601sup1.cif

Structure factors: contains datablock(s) I. DOI: 10.1107/S1600536813013640/su2601Isup2.hkl

Supplementary material file. DOI: 10.1107/S1600536813013640/su2601Isup3.cml

Additional supplementary materials:  crystallographic information; 3D view; checkCIF report

## Figures and Tables

**Table 1:** Hydrogen-bond geometry (Å, °)

*D*—H⋯*A*	*D*—H	H⋯*A*	*D*⋯*A*	*D*—H⋯*A*
C4—H4⋯N2^i^	0.93	2.62	3.500 (3)	158

Symmetry code: (i) 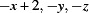.

**Fig. 1.**
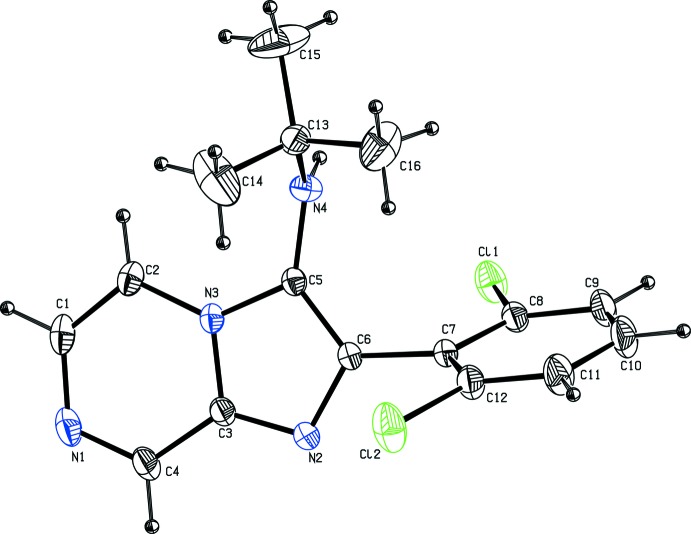
The molecular structure of the title molecule, with atom labelling. Displacement ellipsoids are drawn at the 30% probability level.

**Fig. 2.**
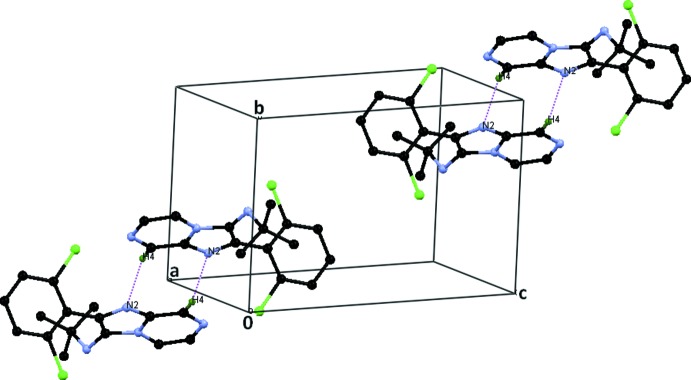
The crystal packing of the title compound viewed along the a axis. The hydrogen bonds are sown as dashed lines (see Table 1 for details; H-atoms not involved in H-bonds have been excluded for clarity).

## supplementary crystallographic information

### Comment

The pyrazine ring system is a useful structural element in medicinal chemistry and has found broad applications in drug development such as antiproliferative agents (Dubinina *et al.*, 2006), potent CXCR3 antagonists (Du *et al.*, 2009), CB1 antagonists (Ellsworth *et al.*, 2007) and c-Src inhibitors (Mukaiyama *et al.*, 2007). On-going structural studies of heterocyclic N-containing derivatives (Nasir *et al.*, 2010) are motivated by an investigation of their fluorescence properties (Kawai *et al.*, 2001; Abdullah, 2005). For multidrug-resistant Tuberculosis (Dooley *et al.*, 1992), antifungal and antimycobacterial activity (Banfi *et al.* 2006) and bactericidal effects (Jackson *et al.* 2000), the use of imidazole based compounds were reported. In view of the different applications of this class of compounds, we have undertaken a single-crystal structure determination of the title compound.

In the titled compound, Fig.1, the imidazole ring (N2/N3/C3/C5/C6) makes a dihedral angle of 1.06 (9)° with the pyrazine ring (N1/N3/C1-C4), and a dihedral angle of 70.01 (1)° with the phenyl ring (C7-C12). The dihedral angle between the pyrazine ring and the phenyl ring is 69.54 (1)°. The chlorine atoms Cl1 and Cl2 attached to the phenyl ring deviate by -0.0472 (6)Å and 0.0245 (8)Å.

In the crystal, molecules are linked via pairs of C—H···N hydrogen bonds forming inversion dimers (Table 1 and Fig.2).

### Experimental

2-aminopyrazine (1.0 mmol) was placed in oven-dried round bottom flask, dissolved in EtOH (5.0 mL) and stirred at room temperature. 2,6-dichlorobenzaldehyde (1.0 mmol), tert-butyl isocyanide (1.0 mmol) and Iodine (2.0 mol%) were added together and the mixture stirred, progress of the reaction was monitored by using TLC, at room temperature for one hour. The reaction mixture was concentrated under reduced pressure and the crude product was partitioned between EtOAc and water. The organic phase was separated, and the residual product in the aqueous phase was extracted with EtOAc (2 × 10 mL). The combined organic extract was dried over anhydrous Na_2_SO_4_, filtered, concentrated and purified using column chromatography (silica gel 60-120 mesh, elutent: 5% EtOAc in hexane).M.p: 421 - 423 k, IR (KBr, cm^-1^): 3353 (NH). After two weeks a colourless crystalline solid separated out. It was washed with a minimum amount of ethanol and then dried in a vacuum oven; a crystal was chosen for X-ray diffraction studies from this sample.

### Refinement

The H atoms were placed in calculated positions and refined as riding atoms: C—H = 0.93 and 0.96 Å for CH and CH_3_ H atoms, respectively, with *U*_iso_(H) = 1.5*U*_eq_(C-methyl) and = 1.2*U*_eq_(C) for other H atoms.

### Crystal data

**Table:**

C_16_H_16_Cl_2_N_4_	*Z* = 2
*M**_r_* = 335.23	*F*(000) = 348
Triclinic, *P*1	*D*_x_ = 1.336 Mg m^−^^3^
Hall symbol: -P 1	Mo *K*α radiation, λ = 0.71073 Å
*a* = 8.1482 (4) Å	Cell parameters from 3438 reflections
*b* = 9.8553 (5) Å	θ = 1.8–26.5°
*c* = 11.5265 (6) Å	µ = 0.39 mm^−^^1^
α = 93.218 (2)°	*T* = 293 K
β = 99.320 (3)°	Block, colourless
γ = 113.026 (2)°	0.30 × 0.25 × 0.20 mm
*V* = 833.31 (7) Å^3^	

### Data collection

**Table:**

Bruker SMART APEXII area-detector diffractometer	3438 independent reflections
Radiation source: fine-focus sealed tube	2941 reflections with *I* > 2σ(*I*)
Graphite monochromator	*R*_int_ = 0.026
ω and φ scans	θ_max_ = 26.5°, θ_min_ = 1.8°
Absorption correction: multi-scan (*SADABS*; Bruker, 2008)	*h* = −10→10
*T*_min_ = 0.892, *T*_max_ = 0.926	*k* = −12→12
12583 measured reflections	*l* = −14→14

### Refinement

**Table:**

Refinement on *F*^2^	Secondary atom site location: difference Fourier map
Least-squares matrix: full	Hydrogen site location: inferred from neighbouring sites
*R*[*F*^2^ > 2σ(*F*^2^)] = 0.040	H-atom parameters constrained
*wR*(*F*^2^) = 0.120	*w* = 1/[σ^2^(*F*_o_^2^) + (0.0616*P*)^2^ + 0.252*P*] where *P* = (*F*_o_^2^ + 2*F*_c_^2^)/3
*S* = 1.05	(Δ/σ)_max_ < 0.001
3438 reflections	Δρ_max_ = 0.34 e Å^−^^3^
203 parameters	Δρ_min_ = −0.40 e Å^−^^3^
0 restraints	Extinction correction: *SHELXL97* (Sheldrick, 2008), Fc^*^=kFc[1+0.001xFc^2^λ^3^/sin(2θ)]^-1/4^
Primary atom site location: structure-invariant direct methods	Extinction coefficient: 0.029 (4)

### Special details

**Table:**

Geometry. All esds (except the esd in the dihedral angle between two l.s. planes) are estimated using the full covariance matrix. The cell esds are taken into account individually in the estimation of esds in distances, angles and torsion angles; correlations between esds in cell parameters are only used when they are defined by crystal symmetry. An approximate (isotropic) treatment of cell esds is used for estimating esds involving l.s. planes.
Refinement. Refinement of F^2^ against ALL reflections. The weighted R-factor wR and goodness of fit S are based on F^2^, conventional R-factors R are based on F, with F set to zero for negative F^2^. The threshold expression of F^2^ > 2sigma(F^2^) is used only for calculating R-factors(gt) etc. and is not relevant to the choice of reflections for refinement. R-factors based on F^2^ are statistically about twice as large as those based on F, and R- factors based on ALL data will be even larger.

### Fractional atomic coordinates and isotropic or equivalent isotropic displacement parameters (Å^2^)

**Table:**

	*x*	*y*	*z*	*U*_iso_*/*U*_eq_	
C1	0.8515 (3)	0.3445 (2)	−0.14305 (16)	0.0590 (5)	
H1	0.8278	0.3957	−0.2044	0.071*	
C2	0.8018 (2)	0.3676 (2)	−0.04035 (15)	0.0490 (4)	
H2	0.7462	0.4327	−0.0307	0.059*	
C3	0.9215 (2)	0.19622 (19)	0.03479 (14)	0.0428 (4)	
C4	0.9674 (3)	0.1803 (2)	−0.07708 (15)	0.0573 (5)	
H4	1.0238	0.1165	−0.0894	0.069*	
C5	0.8067 (2)	0.29067 (17)	0.16413 (13)	0.0374 (3)	
C6	0.8760 (2)	0.19427 (17)	0.21161 (13)	0.0386 (3)	
C7	0.8730 (2)	0.15258 (17)	0.33280 (13)	0.0412 (4)	
C8	0.9765 (2)	0.25222 (19)	0.43377 (14)	0.0466 (4)	
C9	0.9642 (3)	0.2172 (2)	0.54712 (16)	0.0618 (5)	
H9	1.0343	0.2872	0.6126	0.074*	
C10	0.8473 (4)	0.0779 (3)	0.56200 (17)	0.0737 (6)	
H10	0.8378	0.0534	0.6381	0.088*	
C11	0.7438 (4)	−0.0259 (2)	0.46540 (18)	0.0711 (6)	
H11	0.6652	−0.1206	0.4757	0.085*	
C12	0.7582 (3)	0.0125 (2)	0.35278 (16)	0.0543 (4)	
C13	0.5310 (2)	0.3227 (2)	0.20642 (16)	0.0529 (4)	
C14	0.4269 (3)	0.2237 (5)	0.0930 (3)	0.1323 (15)	
H14A	0.4406	0.2817	0.0282	0.198*	
H14B	0.3004	0.1768	0.0967	0.198*	
H14C	0.4727	0.1489	0.0811	0.198*	
C15	0.4854 (5)	0.4564 (4)	0.2163 (4)	0.1293 (15)	
H15A	0.5521	0.5183	0.2900	0.194*	
H15B	0.3572	0.4243	0.2137	0.194*	
H15C	0.5178	0.5118	0.1516	0.194*	
C16	0.4855 (4)	0.2406 (4)	0.3101 (3)	0.1010 (10)	
H16A	0.5025	0.1498	0.3006	0.151*	
H16B	0.3611	0.2180	0.3144	0.151*	
H16C	0.5639	0.3012	0.3817	0.151*	
N1	0.9338 (2)	0.2515 (2)	−0.16355 (13)	0.0635 (4)	
N2	0.9474 (2)	0.13643 (16)	0.13275 (12)	0.0466 (3)	
N3	0.83689 (17)	0.29093 (14)	0.04973 (11)	0.0386 (3)	
N4	0.72957 (19)	0.38082 (15)	0.20777 (13)	0.0471 (3)	
H4A	0.7978	0.4719	0.2361	0.057*	
Cl1	1.12956 (7)	0.42849 (6)	0.41790 (4)	0.0670 (2)	
Cl2	0.62201 (10)	−0.11935 (6)	0.23322 (5)	0.0897 (3)	

### Atomic displacement parameters (Å^2^)

**Table:**

	*U*^11^	*U*^22^	*U*^33^	*U*^12^	*U*^13^	*U*^23^
C1	0.0591 (11)	0.0737 (12)	0.0376 (9)	0.0192 (9)	0.0069 (8)	0.0206 (8)
C2	0.0468 (9)	0.0563 (10)	0.0433 (9)	0.0191 (8)	0.0079 (7)	0.0192 (7)
C3	0.0458 (8)	0.0510 (9)	0.0335 (8)	0.0213 (7)	0.0092 (6)	0.0038 (6)
C4	0.0675 (12)	0.0724 (12)	0.0367 (9)	0.0316 (10)	0.0160 (8)	0.0033 (8)
C5	0.0377 (7)	0.0415 (7)	0.0325 (7)	0.0145 (6)	0.0092 (6)	0.0062 (6)
C6	0.0440 (8)	0.0432 (8)	0.0302 (7)	0.0191 (6)	0.0081 (6)	0.0047 (6)
C7	0.0520 (9)	0.0461 (8)	0.0309 (7)	0.0244 (7)	0.0101 (6)	0.0076 (6)
C8	0.0552 (10)	0.0493 (9)	0.0363 (8)	0.0215 (8)	0.0109 (7)	0.0055 (7)
C9	0.0847 (14)	0.0649 (11)	0.0316 (8)	0.0274 (10)	0.0090 (8)	0.0024 (8)
C10	0.1114 (18)	0.0719 (13)	0.0364 (10)	0.0311 (13)	0.0228 (11)	0.0187 (9)
C11	0.1029 (17)	0.0547 (11)	0.0495 (11)	0.0206 (11)	0.0241 (11)	0.0198 (9)
C12	0.0718 (12)	0.0473 (9)	0.0394 (9)	0.0199 (8)	0.0099 (8)	0.0067 (7)
C13	0.0517 (10)	0.0700 (11)	0.0510 (10)	0.0342 (9)	0.0207 (8)	0.0183 (9)
C14	0.0456 (13)	0.225 (4)	0.092 (2)	0.0308 (19)	0.0034 (13)	−0.041 (2)
C15	0.121 (3)	0.123 (3)	0.221 (4)	0.095 (2)	0.105 (3)	0.087 (3)
C16	0.0738 (16)	0.136 (2)	0.113 (2)	0.0448 (17)	0.0477 (16)	0.076 (2)
N1	0.0727 (11)	0.0839 (12)	0.0337 (8)	0.0292 (9)	0.0157 (7)	0.0109 (7)
N2	0.0590 (8)	0.0559 (8)	0.0350 (7)	0.0324 (7)	0.0130 (6)	0.0071 (6)
N3	0.0375 (6)	0.0449 (7)	0.0317 (6)	0.0144 (5)	0.0071 (5)	0.0083 (5)
N4	0.0487 (8)	0.0432 (7)	0.0548 (8)	0.0213 (6)	0.0177 (6)	0.0064 (6)
Cl1	0.0707 (3)	0.0591 (3)	0.0495 (3)	0.0047 (2)	0.0110 (2)	0.0012 (2)
Cl2	0.1200 (5)	0.0537 (3)	0.0557 (3)	−0.0004 (3)	0.0043 (3)	−0.0005 (2)

### Geometric parameters (Å, º)

**Table:**

C1—C2	1.347 (3)	C9—H9	0.9300
C1—N1	1.362 (3)	C10—C11	1.375 (3)
C1—H1	0.9300	C10—H10	0.9300
C2—N3	1.376 (2)	C11—C12	1.383 (3)
C2—H2	0.9300	C11—H11	0.9300
C3—N2	1.327 (2)	C12—Cl2	1.7307 (19)
C3—N3	1.378 (2)	C13—N4	1.487 (2)
C3—C4	1.417 (2)	C13—C14	1.494 (3)
C4—N1	1.304 (3)	C13—C16	1.496 (3)
C4—H4	0.9300	C13—C15	1.506 (3)
C5—C6	1.378 (2)	C14—H14A	0.9600
C5—N3	1.3800 (19)	C14—H14B	0.9600
C5—N4	1.388 (2)	C14—H14C	0.9600
C6—N2	1.3650 (19)	C15—H15A	0.9600
C6—C7	1.479 (2)	C15—H15B	0.9600
C7—C12	1.389 (2)	C15—H15C	0.9600
C7—C8	1.391 (2)	C16—H16A	0.9600
C8—C9	1.379 (2)	C16—H16B	0.9600
C8—Cl1	1.7383 (18)	C16—H16C	0.9600
C9—C10	1.370 (3)	N4—H4A	0.8600
			
C2—C1—N1	124.75 (17)	C11—C12—Cl2	117.84 (15)
C2—C1—H1	117.6	C7—C12—Cl2	119.48 (13)
N1—C1—H1	117.6	N4—C13—C14	109.91 (16)
C1—C2—N3	117.18 (17)	N4—C13—C16	111.05 (16)
C1—C2—H2	121.4	C14—C13—C16	110.3 (3)
N3—C2—H2	121.4	N4—C13—C15	106.32 (19)
N2—C3—N3	111.51 (13)	C14—C13—C15	110.4 (3)
N2—C3—C4	131.36 (16)	C16—C13—C15	108.7 (2)
N3—C3—C4	117.12 (15)	C13—C14—H14A	109.5
N1—C4—C3	122.75 (18)	C13—C14—H14B	109.5
N1—C4—H4	118.6	H14A—C14—H14B	109.5
C3—C4—H4	118.6	C13—C14—H14C	109.5
C6—C5—N3	104.18 (13)	H14A—C14—H14C	109.5
C6—C5—N4	134.65 (14)	H14B—C14—H14C	109.5
N3—C5—N4	121.10 (13)	C13—C15—H15A	109.5
N2—C6—C5	112.36 (13)	C13—C15—H15B	109.5
N2—C6—C7	122.10 (13)	H15A—C15—H15B	109.5
C5—C6—C7	125.53 (13)	C13—C15—H15C	109.5
C12—C7—C8	115.75 (14)	H15A—C15—H15C	109.5
C12—C7—C6	121.53 (14)	H15B—C15—H15C	109.5
C8—C7—C6	122.64 (14)	C13—C16—H16A	109.5
C9—C8—C7	122.83 (17)	C13—C16—H16B	109.5
C9—C8—Cl1	117.98 (14)	H16A—C16—H16B	109.5
C7—C8—Cl1	119.18 (12)	C13—C16—H16C	109.5
C10—C9—C8	119.10 (18)	H16A—C16—H16C	109.5
C10—C9—H9	120.4	H16B—C16—H16C	109.5
C8—C9—H9	120.4	C4—N1—C1	117.39 (16)
C9—C10—C11	120.58 (18)	C3—N2—C6	104.55 (13)
C9—C10—H10	119.7	C2—N3—C3	120.81 (14)
C11—C10—H10	119.7	C2—N3—C5	131.78 (14)
C10—C11—C12	119.06 (19)	C3—N3—C5	107.39 (12)
C10—C11—H11	120.5	C5—N4—C13	121.18 (14)
C12—C11—H11	120.5	C5—N4—H4A	119.4
C11—C12—C7	122.66 (17)	C13—N4—H4A	119.4
			
N1—C1—C2—N3	−0.2 (3)	C8—C7—C12—Cl2	179.44 (13)
N2—C3—C4—N1	−178.52 (19)	C6—C7—C12—Cl2	2.7 (2)
N3—C3—C4—N1	0.4 (3)	C3—C4—N1—C1	0.2 (3)
N3—C5—C6—N2	0.31 (18)	C2—C1—N1—C4	−0.3 (3)
N4—C5—C6—N2	−176.49 (17)	N3—C3—N2—C6	0.44 (19)
N3—C5—C6—C7	−178.54 (14)	C4—C3—N2—C6	179.42 (19)
N4—C5—C6—C7	4.7 (3)	C5—C6—N2—C3	−0.46 (19)
N2—C6—C7—C12	−71.1 (2)	C7—C6—N2—C3	178.43 (15)
C5—C6—C7—C12	107.6 (2)	C1—C2—N3—C3	0.8 (2)
N2—C6—C7—C8	112.39 (19)	C1—C2—N3—C5	178.89 (16)
C5—C6—C7—C8	−68.9 (2)	N2—C3—N3—C2	178.22 (14)
C12—C7—C8—C9	−1.6 (3)	C4—C3—N3—C2	−0.9 (2)
C6—C7—C8—C9	175.10 (17)	N2—C3—N3—C5	−0.26 (18)
C12—C7—C8—Cl1	177.99 (13)	C4—C3—N3—C5	−179.40 (15)
C6—C7—C8—Cl1	−5.3 (2)	C6—C5—N3—C2	−178.28 (16)
C7—C8—C9—C10	0.9 (3)	N4—C5—N3—C2	−0.9 (3)
Cl1—C8—C9—C10	−178.66 (18)	C6—C5—N3—C3	−0.03 (16)
C8—C9—C10—C11	0.2 (4)	N4—C5—N3—C3	177.31 (14)
C9—C10—C11—C12	−0.5 (4)	C6—C5—N4—C13	−87.5 (2)
C10—C11—C12—C7	−0.3 (4)	N3—C5—N4—C13	96.14 (18)
C10—C11—C12—Cl2	−178.48 (19)	C14—C13—N4—C5	−40.2 (3)
C8—C7—C12—C11	1.3 (3)	C16—C13—N4—C5	82.2 (2)
C6—C7—C12—C11	−175.48 (19)	C15—C13—N4—C5	−159.7 (2)

### Hydrogen-bond geometry (Å, º)

**Table:**

*D*—H···*A*	*D*—H	H···*A*	*D*···*A*	*D*—H···*A*
C4—H4···N2^i^	0.93	2.62	3.500 (3)	158

Symmetry code: (i) −*x*+2, −*y*, −*z*.
